# Comparison of Broth Disk Elution and Broth Microdilution Methods for Colistin Susceptibility Testing in Carbapenem-Resistant Enterobacteriaceae and Pseudomonas aeruginosa

**DOI:** 10.7759/cureus.91811

**Published:** 2025-09-08

**Authors:** Priyanka M Chandankhede, Rajesh P Karyakarte, Ashish Sadafale, Prashant Patil, Rashmita Das, Sushma Yanamandra, Meghna Palewar

**Affiliations:** 1 Microbiology, B. J. Government Medical College & Sassoon General Hospitals, Pune, IND

**Keywords:** broth microdilution method, carbapenamase resistant pseudomonas aeruginosa (crpa), carbapenem-resistant enterobacteriaceae (cre), colistin broth disk elution, colistin resistance

## Abstract

Background: The increasing prevalence of carbapenem-resistant *Enterobacteriaceae* (CRE) and *Pseudomonas aeruginosa* (CRPA) poses a significant challenge to clinical management, particularly in resource-limited settings. Colistin remains one of the last-resort antibiotics for treating these infections; however, its accurate susceptibility testing remains technically challenging. The Broth Microdilution (BMD) method, though recommended as the reference standard, is labor-intensive and not feasible for routine use in most laboratories. This study aims to evaluate the Colistin Broth Disk Elution (CBDE) method as a practical alternative to BMD.

Materials and methods: This cross-sectional study was conducted over 18 months (December 2022 to June 2024) at a tertiary care microbiology laboratory in India. A total of 120 non-duplicate CRE and CRPA isolates from clinical specimens were randomly selected and tested for colistin susceptibility using both BMD and CBDE methods. MIC interpretations were based on CLSI 2024 guidelines. Categorical agreement (CA), essential agreement (EA), and error rates were analyzed as per CLSI 2015 recommendations.

Results: Out of 120 carbapenem-resistant isolates included in the study, 92 (76.67%) were CRE and 28 (23.33%) were CRPA. Colistin resistance was detected in six (5%) and four (3.33%) isolates by BMD and CBDE, respectively. CBDE showed a CA of 98.3% (95% confidence Interval: 94.1%-99.8%) and EA of 100% when compared to BMD, with only two minor errors (1.6%) and no major or very major errors.

Conclusion: CBDE demonstrated high categorical agreement with BMD and minimal error and offers a simple, cost-effective alternative for routine colistin susceptibility testing, especially in low-resource settings where BMD is not feasible.

## Introduction

The emergence and global spread of carbapenem-resistant *Enterobacteriaceae* (CRE) and carbapenem-resistant *Pseudomonas aeruginosa* (CRPA) have become a critical threat to public health, complicating both therapeutic strategies and infection control efforts. *Enterobacteriaceae*, commonly found in the environment and as part of the normal human gut flora, account for over 70% of Gram-negative infections. *P. aeruginosa*, a non-fermenting obligate aerobe, on the other hand, is recognized as a major cause of nosocomial infections [1].

Carbapenems, often reserved for treating severe infections caused by multidrug-resistant Gram-negative bacteria, are increasingly rendered ineffective due to the rise of carbapenem resistance. CRE are particularly concerning, as they often harbor mechanisms conferring resistance not only to carbapenems but to multiple other antibiotic classes, resulting in limited treatment options and increased morbidity and mortality among hospitalized patients [2].

Colistin, a polymyxin antibiotic of last resort, retains activity against both *Enterobacteriaceae* and *Pseudomonas aeruginosa*. However, its clinical utility is constrained by significant nephrotoxicity and challenges associated with accurate in-vitro susceptibility testing [3]. The drug’s strong cationic nature leads to poor diffusion in agar-based methods and adsorption to laboratory plastics, making conventional susceptibility testing unreliable [4,5].

To address these challenges, both the Clinical and Laboratory Standards Institute (CLSI) and the European Committee on Antimicrobial Susceptibility Testing (EUCAST) recommend broth microdilution (BMD) as the reference method for Colistin susceptibility testing. Despite its accuracy, BMD is labor-intensive and resource-demanding, limiting its routine use in many diagnostic laboratories. In response, alternative methods such as the colistin disk elution (CDE) test and Colistin agar test have been proposed and evaluated by expert working groups [5].

With the above background, the present study aims to assess the colistin susceptibility patterns of CRE and CRPA isolates using both the broth microdilution (BMD) and disk elution methods.

## Materials and methods

This hospital-based cross-sectional study was conducted over 18 months, from December 2022 to June 2024, in the Department of Microbiology at Byramjee Jeejeebhoy Government Medical College (BJGMC), Pune, Maharashtra, India.

Ethical considerations

Ethical approval for the study was obtained from the Institutional Ethics Committee, BJGMC, Pune (Approval No. *BJGMC/IEC/Pharmac/D-1022167-167*). Informed consent was obtained from all patients prior to inclusion of the samples. No patient identifiable information was disclosed during the study. All study data were de-identified before analysis.

Sample types and isolate selection

The study included clinical isolates of carbapenem-resistant *Enterobacteriaceae* and *Pseudomonas aeruginosa* obtained from various sample types, including blood, endotracheal aspirates, body fluids (such as pleural, ascitic, and cerebrospinal fluid), pus, and wound swabs. These samples originated from patients attending outpatient departments (OPD), inpatient wards (IPD), and intensive care units (ICUs). Isolates recovered from urine, stool, and sputum samples were excluded, along with genera belonging to the *Morganellaceae* family (e.g., *Proteus*, *Providencia*, *Morganella*) due to their intrinsic resistance to colistin. Repeated isolates from the same patient were also excluded to avoid duplication. Clinical isolates meeting the inclusion criteria were identified and subjected to further analysis.

Phenotypic identification and antimicrobial susceptibility testing

Preliminary identification of *Enterobacteriaceae *and *Pseudomonas aeruginosa* isolates was performed based on colony morphology on blood agar and MacConkey agar, along with standard presumptive tests including Gram staining, catalase, oxidase, and motility tests. Following preliminary identification, speciation was confirmed using a series of standard biochemical tests. Antibiotic susceptibility testing was performed using the Kirby-Bauer disk diffusion method, following standard microbiological procedures. Zones of inhibition for various antibiotics (other than colistin) tested were measured, and results were interpreted according to CLSI M100 (34th Edition, 2024) guidelines [6]. Carbapenem resistance was defined as the absence of a clear inhibition zone or a meropenem (10 µg) zone diameter ≤ 15 mm on disk diffusion.

Colistin susceptibility testing

Colistin susceptibility was studied for all isolates of carbapenem-resistant *Enterobacteriaceae* and *Pseudomonas aeruginosa* using both the Broth Microdilution (BMD) method (the gold-standard method) and the Colistin Broth Disk Elution (CBDE) method. Both tests were performed in parallel. Quality control for BMD and CBDE was performed using *P. aeruginosa* ATCC 27853 and *E. coli* ATCC 25922.

Colistin broth microdilution (BMD) method

Colistin BMD was performed as per the standard operating procedure of the AMR Containment Programme (NCDC, India, January 2023), which follows CLSI M100 (32nd Edition, 2022) recommendations for clinical reporting of MIC values for in vitro broth microdilution of colistin sulfate [7]. A stock solution (1,000 μg/mL) was prepared by dissolving 10 mg of colistin sulfate powder (633 μg/mg; Sigma-Aldrich, USA) in 6.33 mL of sterile water. Working solutions were prepared by four-fold preparation and two-fold serial dilutions to achieve concentrations ranging from 16 to 0.03 μg/mL in microtiter wells.

A standardized inoculum of 0.5 McFarland (~ 1.5 x 10^8^ CFU/mL) was prepared by suspending the 3-5 well-isolated colonies from the 18- to 24-hour culture plate, in sterile saline using the direct colony suspension method. The inoculum density was matched to a 0.5 McFarland BaSO₄ turbidity standard. For inoculation in microtiter plates, a bacterial concentration of ~5 × 104 CFU/well was obtained by diluting the 0.5 McFarland suspension 1:75 times by adding 10 μL of suspension to 740 μL of autoclaved MHB medium.

**Figure 1:**
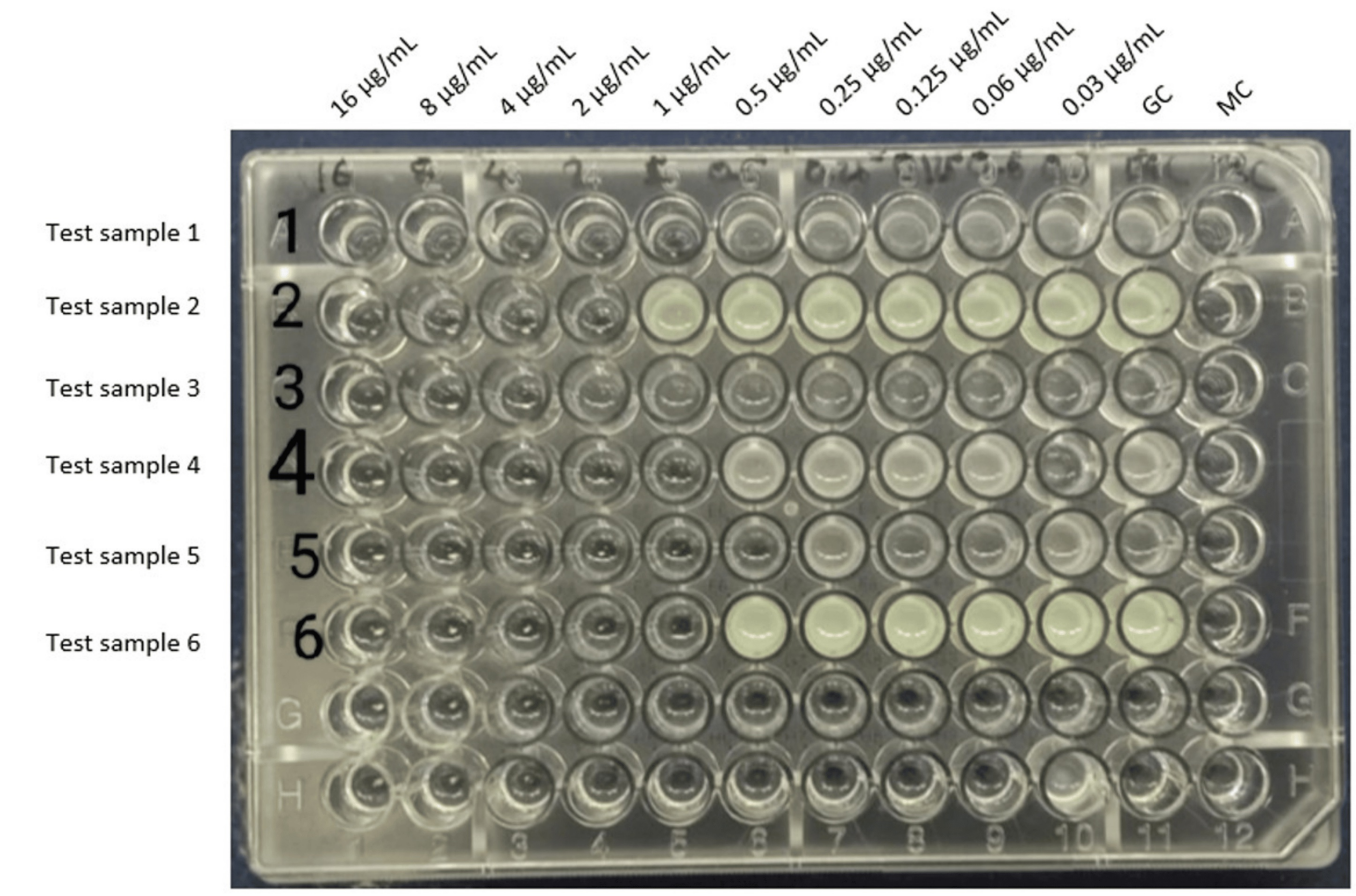
Colistin broth microdilution (BMD) method GC: Growth control, MC: Media control

For each test well (final volume 100 μL), 50 μL of Cation-Adjusted Mueller-Hinton Broth (CAMHB), 25 μL of colistin working solution, and 25 μL of diluted inoculum were added. Growth control wells contained 75 μL CAMHB and 25 μL inoculum, while media control wells contained 100 μL CAMHB without inoculum (Figure 1). Within 15 minutes of inoculation, microtiter plates were incubated at 35 ± 2°C in an ambient air incubator for 16-20 hours for *Enterobacteriaceae* and *Pseudomonas aeruginosa*.

As part of inoculum-density quality control, colony counts were performed on randomly selected cultures. Immediately after inoculation, 10 µL from the growth-control well was diluted 1:1000 in sterile saline, mixed, and 100 µL was spread on tryptic soy or nutrient agar. The plates were incubated at 35 ± 2°C for 16-20 h. The presence of ~50 colonies indicated an inoculum of ~5 × 10^5^ CFU/mL.

The minimum inhibitory concentration (MIC) was recorded as the lowest colistin concentration completely inhibiting visible growth. Following CLSI M100 (34th Edition, 2024) guidelines [6], an MIC ≤ 2 µg/mL was considered as intermediate, and an MIC ≥ 4 µg/mL was considered as resistant.

Colistin broth disk elution (CBDE) method

**Figure 2:**
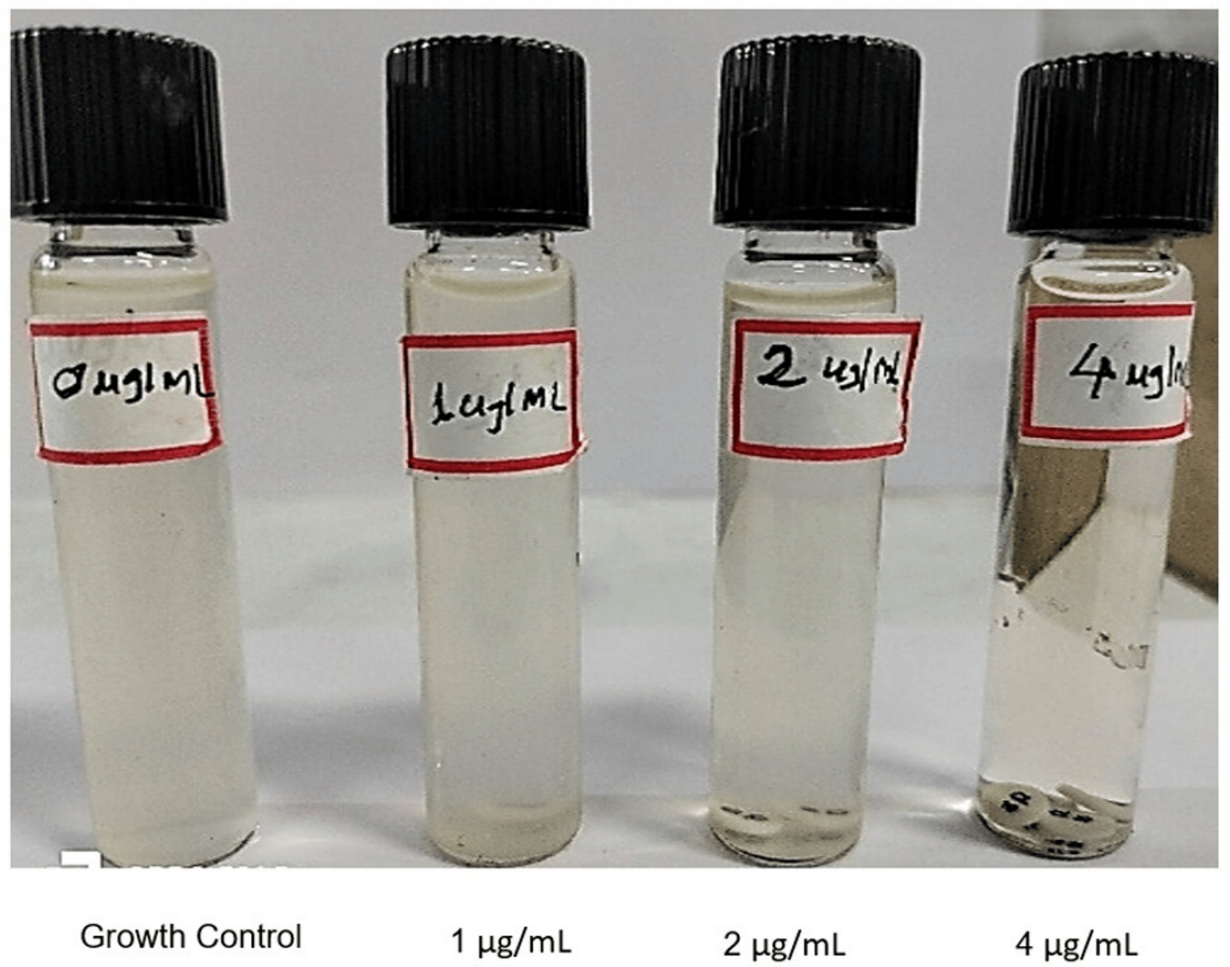
Colistin broth disc elution (CBDE) method

Cation-adjusted Mueller-Hinton Broth (CAMHB, HiMedia Laboratories Pvt. Ltd., Mumbai, India) tubes (10 mL) and colistin sulfate disks (10 µg, HiMedia Laboratories Pvt. Ltd., Mumbai, India) were equilibrated to room temperature. For each isolate, four tubes, control (no colistin) and tubes containing 1, 2, and 4 µg/mL of colistin, were prepared by adding 1, 2, or 4 colistin disks, respectively (Figure 2). The tubes were vortexed and left for 30 minutes at room temperature to allow elution of colistin from the disks.

Inoculum was prepared using fresh colonies from an overnight sheep’s blood agar plate in normal saline and standardizing the turbidity to match that of a McFarland 0.5 standard. A 50 µL of the standardized inoculum (target final concentration ~7.5 × 10^5^ CFU/mL) was added to all tubes. Tubes were vortexed, caps loosened, and incubated at 35oC for 16-20 hours.

Colistin MIC values were determined visually as the lowest concentration that completely inhibited visible bacterial growth. The MICs were interpreted using CLSI M100 (34th Edition, 2024) [6] breakpoints for *P. aeruginosa* and *Enterobacteriaceae*. An MIC ≤ 2 µg/mL was considered intermediate, and an MIC ≥ 4 µg/mL was considered resistant.

Statistical analysis

The MIC values obtained by CBDE were compared to those from the reference BMD method. The agreement between CBDE and BMD was described as essential agreement (EA) and categorical agreement (CA). EA was defined as the CBDE MIC value that is within 1 log_2_ dilution of the reference BMD MIC value, while CA represented the total number of isolates tested using CBDE that yielded a MIC result and the same categorical interpretation as the BMD MIC result (i.e., intermediate and resistant) [8].

Categorical-agreement (CA) discrepancies are further classified into minor errors (mEs), major errors (MEs), and very major errors (VMEs) that reflect MIC differences between the reference broth microdilution (BMD) method and the CBDE method. These rates are reported as percentages, where a VME denotes a false-susceptible result versus BMD, an ME denotes a false-resistant result versus BMD, and an mE occurs when one method reports Intermediate while the other reports susceptible or resistant [8].

According to CLSI M52 guidelines [9], a new method is considered acceptable if CA and EA are ≥ 90%, VME is ≤ 1.5%, ME is ≤ 3%, and mE is ≤ 10%.

## Results

During the study period, a total of 120 non-duplicate, carbapenem-resistant organisms were randomly selected and included for colistin susceptibility testing.

A total of 92 (76.67%) isolates were carbapenem-resistant *Enterobacteriaceae* (CRE), comprising *Klebsiella pneumoniae* (49/92; 53.26%), *Escherichia coli* (23/92; 25%), *Citrobacter* species (11/92; 11.96%), and *Enterobacter* species (9/92; 9.78%). In addition, 28 out of 120 (23.33%) isolates were carbapenem-resistant *Pseudomonas aeruginosa* (CRPA). Table 1 describes the distribution of different carbapenem-resistant organisms isolated from various samples. Wound swabs (43/120, 35.8%), blood (32/120, 26.67%), and pus (31/120, 25.83%) samples accounted for the highest proportion of samples. Surgical wards were the major source of CRE isolates, particularly *K. pneumoniae*. *P. aeruginosa* (CRPA) was predominantly isolated from the "other" category, which included ICU and other wards (Table 2).

**Table 1:** Distribution of bacterial isolates recovered from various clinical specimens

Clinical Specimens	Organisms isolated (n=120)					
	Carbapenem-resistant *Enterobacteriaceae* (CRE) (n=92)				Carbapenem-resistant *Pseudomonas aeruginosa *(CRPA) (n=28)	Total (n=120)
	*Klebsiella pneumoniae* (n=49)	*Escherichia coli* (n=23)	*Citrobacter *species (n=11)	*Enterobacter *species (n=09)		
Wound swab	18 (36.74%)	11 (47.83%)	01 (9.09%)	01 (11.11%)	12 (42.86%)	43 (35.83%)
Blood	15 (30.61%)	08 (34.78%)	00	00	09 (32.14%)	32 (26.67%)
Pus	14 (28.57%)	04 (17.39%)	01 (9.09%)	05 (55.56%)	07 (25%)	31 (25.83%)
CSF	02 (4.08%)	00	02 (18.18%)	01 (11.11%)	00	05 (4.17%)
Others (Ascitic fluid, Pleural fluid, Bronchoalveolar lavage and Tracheal aspirate)	00	00	07 (63.64%)	02 (22.22%)	00	09 (7.5%)

**Table 2:** Distribution of bacterial isolates recovered from various clinical wards

Wards	Organisms isolated (n=120)					
	Carbapenem-resistant *Enterobacteriaceae* (CRE) (n=92)				Carbapenem-resistant *Pseudomonas aeruginosa* (CRPA) (n=28)	Total (n=120)
	*Klebsiella pneumoniae* (n=49)	*Escherichia coli* (n=23)	*Citrobacter* species (n=11)	*Enterobacter* species (n=09)		
Surgery	25 (51.02%)	06 (26.09%)	00	00	04 (14.29%)	35 (29.17%)
Medicine	05 (10.20%)	05 (21.74%)	03 (27.27%)	03 (33.33%)	01 (3.57%)	17 (14.17%)
Orthopedics	10 (20.41%)	00	00	00	05 (17.86%)	15 (12.5%)
Pediatrics	04 (8.16%)	02 (8.69%)	01 (9.09%)	03 (33.33%)	01 (3.57%)	11 (9.17%)
Others (Pulmonary Medicine, Obstetrics and Gynecology, TICU, MICU, NICU, PICU)	05 (10.20%)	10 (43.48%)	07 (63.64%)	03 (33.33%)	17 (60.71%)	42 (35%)

Colistin susceptibility testing results

Colistin MICs for all carbapenem-resistant isolates determined by the BMD method are shown in Table 3. Overall, colistin resistance was observed in 5% (6/120) of the carbapenem-resistant isolates. While seven out of 92 (7.61%) CRE and four out of 28 (14.29%) CRPA isolates exhibited MICs within the intermediate range (≤ 2 µg/mL), five out of 92 (5.43%) CRE and one out of 28 (3.57%) CRPA isolates demonstrated elevated MICs (≥ 4 µg/mL), consistent with colistin resistance.

**Table 3:** Distribution of colistin MIC values by broth microdilution (BMD) method MIC: Minimum inhibitory concentration

Organisms Tested	Colistin MIC Values (μg/mL)						
	0.25	0.5	1	2	4	8	16
	Intermediate				Resistant		
*K. pneumoniae* (n=49)	24 (48.98%)	15 (30.61%)	4 (8.16%)	3 (6.12%)	01 (2.04%)	02 (4.08%)	0
*E. coli* (n=23)	10 (43.48%)	2 (8.70%)	8 (34.78%)	2 (8.70%)	01 (4.35%)	0	0
*Citrobacter* species (n=11)	03 (27.27%)	03 (27.27%)	04 (36.36%)	01 (9.09%)	0	0	0
*Enterobacter* species (n=9)	01 (11.11%)	04 (44.44%)	2 (22.22%)	01 (11.11%)	01 (11.11%)	0	0
*P. aeruginosa* (n=28)	08 (28.57%)	09 (32.14%)	6 (21.43%)	04 (14.29%)	01 (3.57%)	0	0

Similarly, colistin MICs for all carbapenem-resistant isolates determined by the CBDE method are shown in Table 4. Overall, colistin resistance was observed in 3.33% (4/120) of the carbapenem-resistant isolates. While nine out of 92 (9.78%) CRE and four out of 28 (14.29%) CRPA isolates exhibited MICs within the intermediate range (≤ 2 µg/mL), three out of 92 (3.26%) CRE and one out of 28 (3.57%) CRPA isolates demonstrated elevated MICs (≥ 4 µg/mL), consistent with colistin resistance.

**Table 4:** Distribution of colistin MIC values by the colistin broth disk elution (CBDE) method MIC: Minimum inhibitory concentration

Organisms Tested	Colistin MIC Values (μg/mL)		
	1	2	4
	Intermediate		Resistant
*K. pneumoniae* (n=49)	43 (87.76%)	04 (8.16%)	02 (4.08%)
*E. coli* (n=23)	20 (86.96%)	03 (13.04%)	0
*Citrobacter* species (n=11)	10 (90.91%)	01 (9.09%)	0
*Enterobacter* species (n=9)	07 (77.78%)	01 (11.11%)	01 (11.11%)
*P. aeruginosa* (n=28)	23 (82.14%)	04 (14.29%)	01 (3.57%)

Comparison of CBDE MIC values with BMD MIC values

A comparison of the Colistin Broth Disk Elution (CBDE) method with the reference Broth Microdilution (BMD) method is shown in Table 5. For isolates with a CBDE MIC of 1 µg/mL, 100% were categorized as intermediate by BMD, with MICs ranging from 0.25 to 1 µg/mL. For isolates with MICs of 2 µg/mL by CBDE, 11 isolates were confirmed as intermediate by BMD, while two isolates exhibited a one-dilution higher MIC of 4 µg/mL, representing a minor error. All isolates classified as resistant (MIC = 4 µg/mL) by the CBDE method were also resistant by the BMD method, showing full agreement for resistant strains.

**Table 5:** Comparison of colistin susceptibility results by broth disk elution (CBDE) and broth microdilution (BMD) methods * Two isolates exhibited a one-dilution higher MIC of 4 µg/mL, resulting in minor error MIC: Minimum inhibitory concentration

Colistin Broth Disk Elution (CBDE) Method	Colistin MIC (μg/mL) and the count		Colistin Broth Microdilution (BMD) Method							
			Intermediate					Resistant		
			0.125	0.25	0.5	1	2	4	8	16
	Intermediate	1	0	46	33	24	0	0	0	0
		2	0	0	0	0	11	2*	0	0
	Resistant	4	0	0	0	0	0	2	2	0

When comparing CBDE to the reference BMD method, the overall categorical agreement was 98.3% (95% confidence interval: 94.1%-99.8%) (118/120), with a minor error rate of 1.6%, as two isolates were categorized as intermediate by CBDE and resistant by BMD. Essential agreement between CBDE and BMD was 100%, as all MIC values were within ±1 doubling dilution. No major or very major errors were observed during the study.

## Discussion

In the present study, the observed prevalence of CRE was 11%, consistent with reports by Gupta et al. (17-22%) [10], Sharma et al. (19%) [4], and Verma et al. (19%) [11]. CRPA prevalence was 2.5%, similar to the 3.5% reported by Verma et al. [11], though lower than rates reported by Grewal et al. (10-11%) [12] and Bandyopadhyay et al. [13]. *K. pneumoniae *(41%) was the predominant CRE isolate, followed by *E. coli* (13%), consistent with Makled et al. [14]. *P. aeruginosa* accounted for 23.3%, comparable to rates reported by Cheetmala et al. [15]. Most CRE and CRPA isolates in our study originated from wound samples (35.8%) and blood (26.6%), with *K. pneumoniae* being the predominant organism in both sample types. In contrast, studies by Kar et al. [16] and Makled et al. [14] reported urine as the most frequent source of CRE isolates, with *E. coli* identified as the predominant species. Isolates were predominantly recovered from surgical units (35.1%), particularly from postoperative infections and diabetic foot cases, with *K. pneumoniae* again predominating. These findings are in line with findings from Thomas et al. [17], Elandevi et al. [18], and Mohammad et al. [19].

Carbapenemase-producing *Enterobacteriaceae* and *Pseudomonas aeruginosa* are associated with increased morbidity and mortality. Colistin remains one of the last-resort agents for treating serious infections caused by these pathogens [20]. However, the clinical use of colistin has surged nearly 10-fold in recent years, contributing to an alarming rise in colistin resistance. To promote responsible antimicrobial stewardship, microbiology laboratories implement feasible and standardized methods for routine colistin susceptibility testing. Despite this need, many laboratories currently lack the capacity to perform appropriate testing [6,20].

As per national and international guidelines, only broth microdilution (BMD) is accepted as the reference method for colistin susceptibility testing [5]. BMD results showed that 5% of isolates were resistant to colistin. The overall prevalence of colistin resistance (5%) was consistent with Sharma et al. (6.2%) [4] and Rajeshwari et al. (5%) [21], though lower than that reported by Kar et al. (11%) [16]. MIC distribution patterns in our study were similar to those reported by Rajeshwari et al. [21], Sujatha et al. [5], and Vijaypriya et al. [22], with most isolates demonstrating MIC ≤ 1 µg/mL and a small proportion exhibiting MIC ≥ 4 µg/mL.

However, in practice, many laboratories report susceptibility results from automated systems such as VITEK-2, despite the absence of formal endorsement for colistin testing using this platform [19]. Given the complexity of performing manual BMD routinely, there is a critical need to validate more practical alternatives. Following CLSI guidelines 2020, the Colistin Broth Disk Elution (CBDE) method has been widely studied [5]. In the present study, the CBDE method demonstrated excellent performance, with a categorical agreement of 98.3% with BMD, consistent with reports by Sujatha et al. (98%) [5], Sharma et al. (98.4%) [4], Rajeshwari et al. (98%) [21], and Simner et al. (100%) [23]. The minor error rate (1.6%) was also consistent with the literature. Therefore, the Colistin Broth Disk Elution (CBDE) method, first recommended by CLSI in 2020 [20], is a cost-effective, reproducible, and accurate approach for determining colistin minimum inhibitory concentrations (MICs). Given its simplicity and affordability, it serves as a practical alternative to the reference Broth Microdilution (BMD) method, particularly in resource-limited settings, to support appropriate clinical use of this last-resort antibiotic [20].

Limitations of the study

While the present study provides valuable insights into colistin susceptibility testing using the CBDE and BMD methods, a few limitations must be acknowledged while interpreting these findings. First, the study was conducted in a single tertiary care hospital, which limits the generalizability of the findings to other healthcare settings with different patient populations and antimicrobial resistance profiles. Second, only 120 carbapenem-resistant isolates were included, which may not adequately capture the full spectrum of colistin resistance patterns. Third, the study did not perform molecular testing for plasmid-mediated colistin resistance (*mcr* genes), so resistance mechanisms could not be confirmed. In addition, the study excluded urine and stool specimens, which may limit the extrapolation of results to urinary pathogens and gastrointestinal carriage cohorts. These factors should be considered when interpreting the CBDE-BMD agreement and generalizing the findings.

## Conclusions

The emergence of colistin resistance among CRE and CRPA highlights the urgent need for reliable susceptibility testing. While Broth Microdilution (BMD) remains the gold standard, it is labor-intensive and not feasible for routine use in many laboratories. In this limited dataset, the colistin broth disk elution (CBDE) method showed high categorical agreement with BMD and low error rates, making it a viable alternative. Given its simplicity, cost-effectiveness, and reproducibility, CBDE can support antimicrobial stewardship efforts in resource-limited settings. Larger, multi-center evaluations are needed to confirm its performance and generalizability.
